# Are Speeded Tests Unfair? Modeling the Impact of Time Limits on the Gender Gap in Mathematics

**DOI:** 10.1177/00131644221111076

**Published:** 2022-08-16

**Authors:** Andrea H. Stoevenbelt, Jelte M. Wicherts, Paulette C. Flore, Lorraine A. T. Phillips, Jakob Pietschnig, Bruno Verschuere, Martin Voracek, Inga Schwabe

**Affiliations:** 1 Tilburg University, The Netherlands; 2 The Netherlands Institute for Social Research, The Hague, The Netherlands; 3 University of Vienna, Austria; 4 University of Amsterdam, The Netherlands

**Keywords:** gender gap, mathematics, test-taking strategy, stereotype threat, item response theory, missing data

## Abstract

When cognitive and educational tests are administered under time limits, tests may become speeded and this may affect the reliability and validity of the resulting test scores. Prior research has shown that time limits may create or enlarge gender gaps in cognitive and academic testing. On average, women complete fewer items than men when a test is administered with a strict time limit, whereas gender gaps are frequently reduced when time limits are relaxed. In this study, we propose that gender differences in test strategy might inflate gender gaps favoring men, and relate test strategy to stereotype threat effects under which women underperform due to the pressure of negative stereotypes about their performance. First, we applied a Bayesian two-dimensional item response theory (IRT) model to data obtained from two registered reports that investigated stereotype threat in mathematics, and estimated the latent correlation between underlying test strategy (here, completion factor, a proxy for working speed) and mathematics ability. Second, we tested the gender gap and assessed potential effects of stereotype threat on female test performance. We found a positive correlation between the completion factor and mathematics ability, such that more able participants dropped out later in the test. We did not observe a stereotype threat effect but found larger gender differences on the latent completion factor than on latent mathematical ability, suggesting that test strategies affect the gender gap in timed mathematics performance. We argue that if the effect of time limits on tests is not taken into account, this may lead to test unfairness and biased group comparisons, and urge researchers to consider these effects in either their analyses or study planning.

Many studies on gender differences use operational tests that are administered under strict time limits. If an effect of time limits on these tests is not considered, this may impede test fairness, because respondents may receive lower estimates of their ability than under unspeeded circumstances (Lu & Sireci, 2007). This is especially problematic when the effect of the time limits is dissimilar across groups of participants (also referred to as differential speededness, see Dorans et al., 1988; Van der Linden et al., 1999). An example for such a dissimilarity may be a gender gap in how men and women respond to items when these items are administered under a time limit.

The results of earlier research suggest that time limits can help explain gender differences observed in numerical reasoning and cognitive ability tasks (Steinmayr & Spinath, 2017; Voyer, 2011). For example, Steinmayr and Spinath (2017) demonstrated in two experimental studies that men outperformed women in numerical reasoning when a strict time limit (i.e., speeded test) was applied, but this observed gender gap vanished when the time limit was relaxed (i.e., power test was used). The authors hypothesized that women did not become more accurate in their tasks, but rather omitted fewer items when there was no time limit. Similarly, it has been shown that women perform worse compared with men due to time limits on cognitive tasks such as mental rotation tasks (Voyer, 2011). Similar results have been found in the field of academic achievement. For example, on regular university exams, women perform worse under time limits compared with men, by answering fewer items correctly and attempting fewer items in general (De Paola & Gioia, 2016). On the contrary, when respondents are given an *adequate* amount of time to complete a mathematics test, such as standardized examinations like the SAT and GRE, relaxing time limits does not result in a gender-specific increase in performance, but rather in an increase in performance of students with lower mathematics ability, regardless of gender (Bridgeman, McBride, & Monaghan, 2004; Bridgeman, Trapani, & Curley, 2004).

Based on the results of these above-mentioned studies, we expand upon the work by Steinmayr and Spinath (2017) and provide a new contribution to studying the relationship between test-taking strategy (e.g., working speed) and mathematics in relation to gender differences. We hypothesize that observed gender differences on mathematics tests administered under strict time limits may be inflated by gender differences in test-taking strategy related to working speed. Here, we focus on both the constructs of latent mathematics ability and latent working speed, as reflected by the number of items a participant completes before time runs out (i.e., the completion factor). To study gender differences under time limits and to separate whether such differences are observed on the latent mathematics ability or on the latent completion factor, we model these traits explicitly in an item response theory (IRT) model (Glas & Pimentel, 2008). This way, we estimate not only the correlation between ability and completion, but also the contribution of these latent traits to the observed gender gap.

Ability tests with strict time limits are often referred to as speeded tests and can be considered as a combination of speed tests and power tests (Lu & Sireci, 2007). In psychometrics, a power test is a test where participants get enough time to complete all tasks (e.g., items), and their performance is assessed based on the correctness of their answers. A speed test, on the other hand, refers to a test that consists of relatively easy items, but the quantity of items is so high that most participants fail to complete all items within the time limit. Their performance is then assessed based on how many items are completed. In practice, often, a test can neither be seen as a pure power test nor or a pure speed test, but rather as a mixture of both. However, when the time allotted for a test is too strict, tests are labeled as speeded. Speeded tests are tests where most participants cannot answer all items within the time limit, but their performance is still assessed based on the correctness of their answers. Speeded tests are used a lot in practice, for example, in the field of reasoning ability (intelligence) testing (Wilhelm & Schulze, 2002), mental rotation tasks (Voyer, 2011), and in applied investigations into hypothesized stereotype threat effects (Flore, 2018). Stereotype threat theory aims to explain observed group differences in academic achievement, such as those between highly achieving men and women, or between highly achieving Black and White students (Spencer et al., 1999; Steele & Aronson, 1995) by suggesting that performance stereotypes lower the performance of negatively stereotyped groups through different test-taking strategies (Rivardo et al., 2011) and by impeding working memory (Schmader et al., 2008). Many stereotype threat experiments use strict time limits to increase test difficulty (Flore, 2018), which is known to increase construct-irrelevant variance in the test scores (Lu & Sireci, 2007). Therefore, we here focus on the effects of time limits in relation to the gender gap in mathematics, and the potential role of experimentally induced stereotype threats therein.

In the presence of a time limit, scores from participants no longer reflect latent mathematical ability, but also contain a speed component (for an overview, see Lord, 1956; Lu & Sireci, 2007; Partchev et al., 2013). Furthermore, if gender differences exist on this second speed factor, observed gender differences cannot be attributed solely to differences in latent mathematics ability. This speed component, like the underlying latent ability for mathematics, can be interpreted as participants’ tendency to complete items and can be regarded as a latent trait. Glas and Pimentel (2008) proposed that as an indicator for this latent trait, the number of non-reached items of an individual can be used (i.e., the completion factor, labeled as missingness propensity in their account). In other words, not only participants’ mathematical ability, but also their latent speed component (defined as the completion factor) can lead to individual differences in observed scores on a mathematics test. Participants’ completion factor reflects how many items they were able to answer within time limits and thus is a reasonable indicator for their working speed.

## Modeling Nonignorable Missing Data Under Time Limits

To model the completion factor of participants correctly, we used a two-dimensional IRT model that allowed us to directly relate the performance on a mathematics test and the proportion of missingness to respondents’ latent mathematics ability and latent completion factor (Glas & Pimentel, 2008). Traditionally, most experimental studies analyze results using composite scores (e.g., sum scores), for example, using Analysis of (Co)variance (AN(C)OVA) models in stereotype threat data (Wicherts et al., 2005). However, as discussed above, these sum scores cannot uncover why group differences on a sum score are observed and they do not allow a test of measurement invariance (Meredith, 1993) across different groups. Moreover, analyzing composite scores requires some scoring rule about the missing responses in the data that may not be suitable in case of nonignorable missing data, such as under time limits. That is, missingness patterns and ability levels may be correlated (see, e.g., Pohl et al., 2014). As discussed, we used the missing data (e.g., non-reached items) of participants as an indicator for their latent completion factor. These issues can be circumvented by using an IRT framework. Another reason for choosing the IRT framework is that it allowed us to study the potential effect of covariates, for example, gender and stereotype threat condition versus control (De Boeck & Wilson, 2004).

In this study, we investigate whether time limits may (partly) explain gender differences on speeded mathematics tests using data from two large preregistered stereotype threat studies (Flore et al., 2018; Stoevenbelt et al., in principle accepted) as an example.

Stereotype threat theory was originally proposed to explain the performance gap between highly achieving White and Black students (Steele & Aronson, 1995), and a large part of the literature still focuses on this performance gap (Nadler & Clark, 2011). However, stereotype threat theory also proposes an explanation for the gender gap in mathematics performance by stating that women underperform compared with their male peers, not because they are less able than men are, but because they feel the additional pressure of the negative stereotype that women have lower mathematical ability (Spencer et al., 1999). Stereotype threat researchers often use speeded tests to maximize the effect of their manipulation, because researchers expect that negative stereotype threat affects women’s performance only when the mathematics test is difficult enough (Keller, 2007; Spencer et al., 1999). In contrast, we argue that the combination of potential negative effects of a time limit and additional negative effects of stereotype threat on women’s performance will lead to a *double disadvantage* for women (Nguyen et al., 2003; Quinn & Spencer, 2001): We hypothesize that women attempt fewer test items (i.e., show more missing data) in the stereotype threat condition compared with those in the control condition. The hypothesis is supported by the results of a study by Flore (2018) that found the stereotype threat effect to be associated with more missing data, because studies with a large number of missing responses (> 20%) indicated larger stereotype threat effects (Cohen’s *d*s > 0.47) compared with studies with less missing data (0.16 < *d* < 0.29).

Both data sets analyzed in the current study are large enough to fit IRT models and were previously collected in large, preregistered studies on stereotype threat. We included the high school study from Flore et al. (2018; henceforth referred to as high school data) and the preliminary data set of the registered replication report (RRR; Simons et al., 2014) on stereotype threat (Stoevenbelt et al., in principle accepted; university data) of the seminal study by Johns et al. (2005). Both studies employed strict time limits to heighten the difficulty of the mathematics test as is commonly done in experimental studies in this research field

In this study, we address the potential negative effect of imposing a strict time limit on women’s performance on speeded mathematics tests. In other words: Do women show a different test-taking behavior than men, and does this negatively affect their performance? Furthermore, we explore the presence of a double disadvantage among women in the experimental condition of a stereotype threat research study: Is female participants’ performance affected not only by the perceived stereotype threat, but *also* by adopting a less efficient test-taking strategy because of strict time limits?

## Method

To answer our research questions, we applied a multiple Bayesian multidimensional IRT model (Glas & Pimentel, 2008) on data obtained from two large preregistered stereotype threat experiments (Flore et al., 2018; Stoevenbelt et al., in principle accepted), totaling 2,858 participants. We used the following approach: First, we identified whether there is a relationship between latent mathematical ability and the latent completion factor, for which we expected that more able participants drop out later in the test (i.e., a positive correlation between latent mathematical ability and the latent completion factor). Second, using three additional models, we explored the effect of person-specific covariates (gender, stereotype threat condition vs. control, and their interaction). Here, we expected all covariates (and their interaction) to have negative effects on both traits. Our hypotheses and confirmatory analyses were preregistered via the Open Science Framework (https://osf.io/s7j8h).

### Deviations From Preregistration

We preregistered to include the data set from the registered report of Flore et al. (2018) who collected data at Dutch high schools in fall 2016 and spring 2017 (data available upon request, because this data set includes the data of minors). The data comprised 2,126 participants, with a final sample of 2,064 after applying the preregistered exclusion criteria (62 exclusions, 2.9% of all participants), of which 1,028 self-identified as male and 1,036 as female. A full description of the data can be found in our preregistration (https://osf.io/s7j8h). We attempted to estimate the confirmatory models (see the subsection: IRT models) on the 20 items in the high school data set to investigate whether the results found in the university sample would replicate. However, even with a large number of burn-in iterations (i.e., 100,000), none of the three Markov chain Monte Carlo (MCMC) chains did achieve stationarity (i.e., did not approach the joint posterior or target distribution sufficiently), even when more informative priors were used (see Appendix B for a classical test theory approach). This likely has to do with the fact that the data showed little variance in the observed mathematics scores. Eight items had a proportion correct score of above 75%, thus suggesting that students perceived the items as relatively easy. Furthermore, the reliability of scores was relatively low, and amounted, assessed by Cronbach’s alpha, to α = .59. Therefore, only results based on the university sample are reported subsequently.

We preregistered comparisons of Models 1 to 4, using the deviance information criterion (DIC; Spiegelhalter et al., 2002), but decided to omit this test, as the effect of the covariates we added to Models 2 to 4 were null to small.

### University Data

The data were originally collected as part of preregistered large-scale stereotype threat experiments (Stoevenbelt et al., in principle accepted). Part of the data were used for exploratory analyses (*N* = 635, or 79% of the final, confirmatory university sample). Specific sample sizes for the data can be found in Table 1.

**Table 1.** Sample Sizes per Data Set.

Dataset	Women, control group	Women, ST group	Men, control group	Men, ST group	Total
Exploratory data (University samples: Tilburg 1 & 2, Amsterdam)	*n* = 284	*n* = 227	*n* = 69	*n* = 55	*N* = 635
Confirmatory data (University samples: Tilburg 1 & 2, Amsterdam, Vienna)	*n* = 334	*n* = 284	*n* = 95	*n* = 81	*N* = 794

*Note.* ST = stereotype threat.

The first sample (henceforth referred to as the university sample) was collected between fall 2019 and fall 2020, as part of a RRR (data available at https://osf.io/6d4pt). The complete university data comprised four subsets. Two samples were collected at Tilburg University (Netherlands; *n* = 188 [English-speaking, sample 1], *n* = 249 [Dutch-speaking, sample 2]), one sample was collected at the University of Amsterdam (Netherlands, *n* = 198), and one sample was collected at the University of Vienna (Austria, *n* = 159). Eight hundred twenty-two undergraduate participants were tested in groups of 5 to 10 participants and assigned to either an experimental stereotype threat condition or the control condition. The original study featured two control conditions studies, but since we did not expect differences between the respective control conditions (see also Johns et al., 2005), we collapsed the two original control conditions. All participants completed 30 multiple-choice items (with five answer options) within a time limit of 20 minutes. Here, we use 25 of these 30 administered items, which were common to both versions of the tests that were used during the RRR (easy vs. regular version). The tests included items that originated from the Graduate Records Examination (obtained from Brown & Pinel, 2003; Inzlicht & Ben-Zeev, 2000, 2003; Jamieson & Harkins, 2009; Johns et al., 2005; Marx & Roman, 2002; Rivardo et al., 2008; Rydell et al., 2009; Schimel et al., 2004; Thoman et al., 2008). All participants that had been originally excluded (*n* = 28, 3.4%) in the RRR were excluded here as well, following the preregistered exclusion criteria (see https://osf.io/7jhds for the script that was used for data cleaning), resulting in a total sample of 794 students (618 self-identified as female and 176 as male). As part of these exclusion criteria, we excluded all participants who omitted an answer to the gender variable or described their gender as “other.” Because we already had studied the data collected in Tilburg and Amsterdam, the analyses of these data were exploratory (exploratory data: https://osf.io/brsnh; confirmatory data: https://osf.io/6d4pt).

We performed a prior analysis to estimate the statistical power that is necessary to detect a correlation between the two latent traits (mathematical ability and completion factor). Details of this power analysis can be found in the preregistration (see https://osf.io/wgcpz). A sample size of *N* = 635 and a correlation of ρ = .50 between the latent traits was sufficient to consequently estimate a positive covariance between the latent traits. Based on these results, we did not expect any power issues concerning the proposed analyses.

### IRT Models

Following Glas and Pimentel (2008), we explicitly modeled two latent traits: mathematics ability and the completion factor. Under the IRT framework, the probability that a participant answers an item correctly is explained by both the participant’s latent trait value and by characteristics of the item, like its difficulty (Embretson & Reise, 2000). The Glas and Pimentel (2008) model is a two-dimensional parametric IRT model that explicitly incorporates the items left unreached because of the time limit and the responses to answered items separately in two two-parameter logistic models (2PLMs). Here, for every participant a missing data indicator is defined as follows:

(1) $$d_{nk}=\;\{\begin{array}{c} 1\;\;\;\;\;if\;x_{\mathrm{nk}}\;was\;observed;\\ 0\;\;\;\;\;if\;x_{nk}\;was\;the\;first\;response\;of\;a\;sequence\;x_{\mathrm{nk}}=9,\;h=k,\;\ldots K;\\ NA\;\;\;\;\;if\;otherwise. \end{array}$$

where $d_{\mathrm{nk}}$ is defined as the observation on the response indicator for person *n* on item *k* (for the distributional properties the missingness indicator, see Glas & Pimentel, 2008), *K* is the total number of items, and *h* is the item number indicator. To create these completion indicators $d_{\mathrm{nk}}$ for the respondents in the data set, a new variable had to be coded for every participant: All items that the participants attempted (i.e., they circled an answer option) received a score of 1. The first non-reached item of a sequence of not-answered items at the end of the test was scored with 0, and all consecutive items were scored as missing (see our preregistration for two examples, https://osf.io/s7j8h). Skipped items that were missing because they were not attempted by the participants rather than missing due to the time limit were scored as 1 (see Holman & Glas, 2005, for a model that accounts for skipped items). Thus, the model used two different sources of information: (a) the observed responses to the mathematics test and (b) the missingness indicator, resulting in two data points per item (namely, raw answer and missingness indicator) for each respondent. These data are used to model two latent traits: The completion factor ( $\theta_{n0}$ ) reflecting participants’ tendency to attempt more items, and mathematical ability ( $\theta_{n1}$ ) reflecting participants’ mathematical ability to answer completed items correctly.

To model the completion factor, using the data from the missingness indicators, we fitted a Rasch model for $p_{k}(\theta_{n0})$ , where $\beta_{k0}$ is the item difficulty parameter. This parameter reflects the position of an item on the latent ability scale, where $\beta_{k0}$ reflects the value of $\theta_{n0}$ . for a respondent who has a probability of 0.5 to provide an answer to that particular item (completion factor) or to answer the item correctly (for $\theta_{n1}$ mathematics ability) (see Embretson & Reise, 2000). The completion factor, thus, models the “steps” that a respondent takes until they stop answering items (i.e., all items that could be answered before the time limit was reached)

(2) $$p_{k}\left(\theta_{n0}\right)=\;\frac{\exp\left(\theta_{n0}-\beta_{k0}\right)}{1+\;\exp\left(\theta_{n0}-\beta_{k0}\right)}.$$

We restricted $\beta_{k0}$ (with parameter estimate *d_k_*) such that the change in probability is uniform over the test, wherein the first items are more likely to be answered than items later in the test:

(3) $$\beta_{k0}=\;\tau_{0}+\left(k-K\right)\tau_{1}.$$

For every participant, the observed responses (0 = incorrect answer, 1 = correct answer) to reached items were used to estimate the probability of answering the item correctly, given participant’s estimated latent mathematics ability $\theta_{n1}$ . To model the latent trait mathematics ability, we again fitted a Rasch model, where $\beta_{k}$ represents the difficulty parameter of the mathematics items (with parameter estimate *b_k_*).

(4) $$p_{k}\left(\theta_{n1}\right)=\;\frac{\exp\left(\theta_{n1}-\beta_{k1}\right)}{1+\;\exp\left(\theta_{n1}-\beta_{k1}\right)}.$$

**Figure 1.**
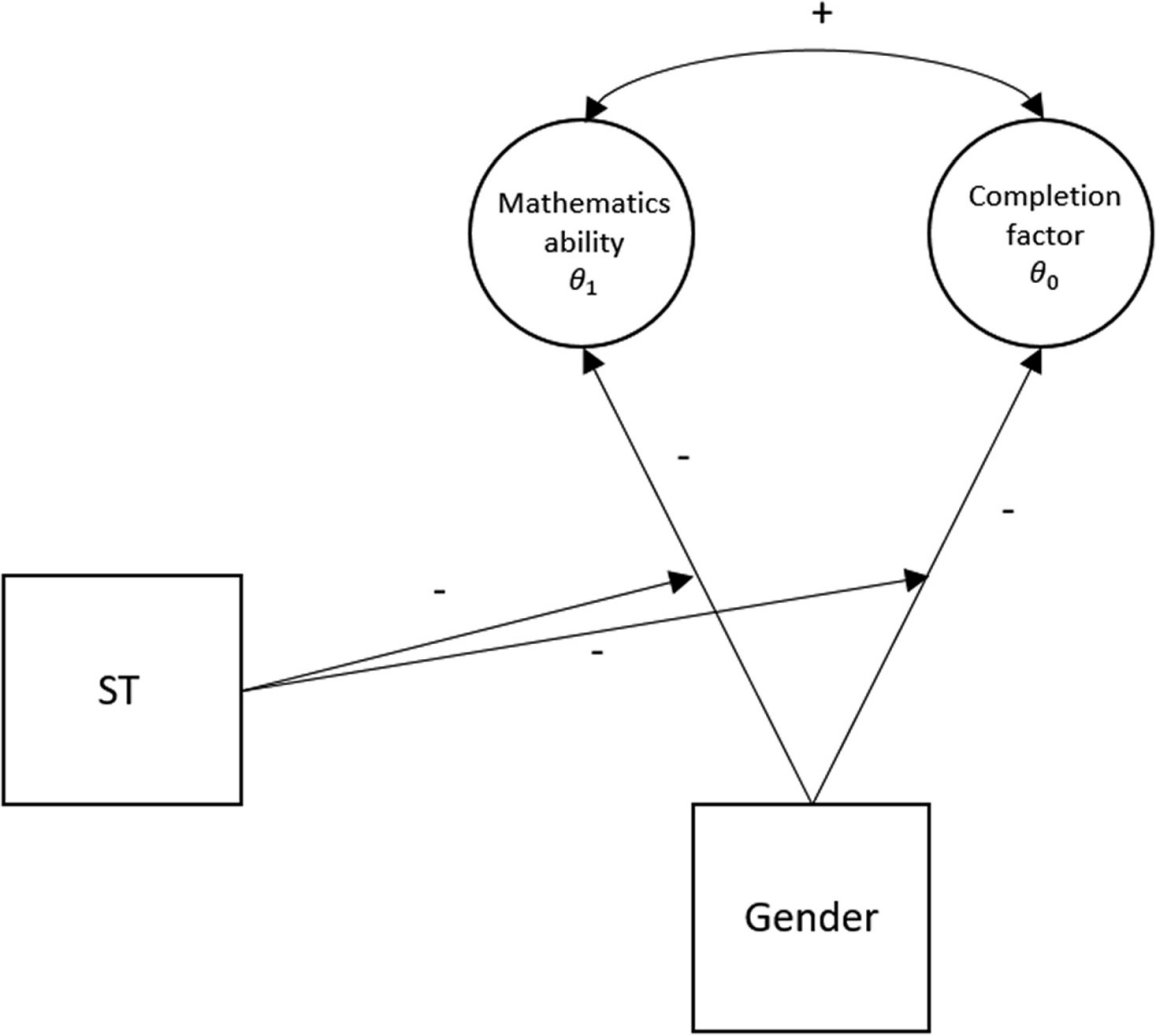
Representation of Model 4, Wherein We Expected a Gender Gap (Dummy Coded, 1 for Women) on Both Latent Traits, With Women Scoring Lower Than Men, and an Additional Negative Effect of the Stereotype Threat Manipulation (Dummy Coded as 1), and a Positive Correlation Between the Two Latent Traits. *Note.* ST = stereotype threat.

To investigate the effect of person-specific covariates on the relationship between missingness and latent ability, we extended the above model (following Glas et al., 2015) by integrating the effects of the covariates gender (1 = women, 0 = men) and condition (1 = stereotype threat, 0 = control). In the first extension (Model 2), the main effect of the gender variable was added; in the second extension, we estimated the main effect of the stereotype threat condition variable (Model 3); and in the last model, both main effects and their interaction were included (Model 4). Model 4 is represented in Figure 1. Finally, we note that here we assumed that the effects of stereotype threat are linear, although there might be scenarios in which this is not the case (for details, see the Discussion section).

### Estimation and Analysis

We fitted four different Bayesian IRT models. Our first model (Model 1) consisted of the model proposed by Glas and Pimentel (2008) which jointly models the relationship between completion factor $\theta_{0}$ and ability $\theta_{1}$ (Glas et al., 2015; Glas & Pimentel, 2008) without taking into account any covariates. In the remaining Models 2 to 4, this basic model was extended by including covariates at the person level (following De Boeck & Wilson, 2004; Glas et al., 2015). All four models were estimated on each confirmatory data set (Vienna data), resulting in a total of eight confirmatory models. We already ran these same four models based on the exploratory data (Tilburg & Amsterdam data) to determine the MCMC settings (assessment of convergence). In each model, we calculated the correlation between the two latent traits based on the obtained variance-covariance matrix.

#### MCMC Estimation

In the Bayesian estimation, our proposed prior distributions (see Priors) of the parameters were updated by the likelihood function of the data sets, resulting in joint posterior distributions for the parameters. In practice, analytical solutions of these posterior distributions are hard to obtain, so we used Gibbs sampling, a MCMC algorithm, to approximate the posterior distributions of all relevant parameter (Geman & Geman, 1984).

For the MCMC estimation, we used the open-source Gibbs sampler JAGS (Plummer, 2003). For further data handling, the statistical programming language R was used (R Core Team, 2021). As an interface from R to JAGS, we used the rjags and coda packages (Plummer et al., 2006, 2019). After an adaptation phase of 25,000 iterations and a burn-in phase of 25,000 iterations for every separate chain, the posterior distribution was based on a total of 60,000 iterations from three separate chains. We use different initial values per chain, drawn from normal (for all parameters except variances) or uniform distributions (only for variances featured in the model), reflecting reasonable parameter values. The same settings were used for all eight models.

#### Convergence

We assessed convergence by inspecting trace plots, as well as computing the Gelman–Rubin convergence diagnostic $\hat{R}$ (Gelman & Rubin, 1992) for all relevant parameters. $\hat{R}$ quantifies the between-chain versus within-chain variance. If $\hat{R}$ is larger than 1, there is evidence that a chain did not converge for a certain parameter (Gelman & Rubin, 1992). We defined $\hat{R}>1.05$ as an indicator for non-convergence. We furthermore inspected Monte Carlo standard errors and effective sample sizes, for which we interpreted a standard error above $SE_{\mathrm{MC}}=0.05$ and an effective sample size below 400 as indication for non-convergence. Details on model convergence can be found in Appendix A.

#### Inference

We calculated posterior means and standard deviations of all parameters of interest (i.e., item parameters estimates *b*_k_ and *d*_k_, the variance-covariance matrix of the latent traits, and the regression coefficients $\beta_{j}$ , $\gamma_{j}$ , and $\delta_{j}$ ), as well as the 95% highest posterior density intervals (HPDI) using the R package BayesTwin (version 1.0; Schwabe, 2017). The 95% HPDI can be interpreted as the Bayesian analogue of a frequentist confidence interval: the influence of a model parameter can be regarded as significant, when the respective interval does not contain zero (with the exception of variances, because their lower bound is zero). Furthermore, the estimated variance-covariance matrix of a respective model was used to calculate the correlation between the two latent traits (i.e., completion factor $\theta_{0}$ and mathematics ability $\theta_{1}$ ).

#### Priors

All priors were chosen such that they can be regarded as relatively uninformative. For the difficulty parameter $d_{k}$ , we defined a linear prior that ensured that the estimate for $d_{k}$ increased for every additional item, of the form $d_{k}=\;\tau_{0}+(k-K)\times \tau_{1}$ , where we placed normal hyperpriors on $\tau_{1}\sim N(0,10)$ , and $\tau_{0}$ was fixed to 1. For the difficulty $b_{k}$ , we defined a normal prior of $b_{k}\sim N(0,\;\sigma_{b})$ , with an hyperprior on the standard deviation of the $b_{k}$ parameters, $\sigma_{b}\sim \mathrm{InvGamma}(.1,.1)$ . For the latent ability estimates, we defined a multivariate normal prior of $\theta_{i}\sim \mathrm{MVN}(0,\tau_{\theta })$ , where we placed a hyperprior on the inverse of the variance-covariance matrix, $\tau_{\theta }$ : $\tau_{\theta }\sim \mathrm{Wishart}(\Omega ,\;\mathrm{NDIM}+1)$ . The means of both latent traits were fixed to 0, in order to identify the model. Finally, for all regression coefficients, we defined normal priors of $\beta_{j},\;\gamma_{j,\;}\;\delta_{j}\sim N(0,\;10)$ .

## Results

Combined over conditions and genders, participants in the university data completed on average 10.5 (out of 25) items and answered on average 6.9 items correctly, resulting in a mean proportion correct of 58% (for descriptive statistics, see Table 2). We report results for the models separately, starting with the model without the covariates gender and stereotype threat.

**Table 2.** Descriptive Statistics per Subsample (University Data).

	Items attempted	Women	Men	Items correct	Women	Men
	Overall			Overall		
Tilburg 1 (ENG)	9.89 (4.52)	9.59 (4.36)	10.88 (4.94)	5.89 (4.08)	5.55 (3.54)	7.05 (5.42)
Tilburg 2 (NL)	10.82 (4.07)	10.48 (3.88)	11.98 (4.71)	6.90 (3.82)	6.67 (3.76)	7.98 (3.92)
Amsterdam	11.66 (5.09)	11.34 (5.10)	13.03 (4.91)	7.92 (4.67)	7.47 (4.63)	9.86 (4.35)
Vienna	9.30 (3.80)	9.06 (3.66)	9.79 (4.08)	6.72 (3.27)	6.49 (3.24)	7.19 (3.30)
All samples	10.50 (4.48)	10.28 (4.38)	11.28 (4.75)	6.88 (4.07)	6.59 (3.93)	7.91 (4.36)

*Note.* Table entries are mean values, with standard deviations (*SD*) in parentheses below.

### Model 1: Estimating the Correlation Between the Completion Factor and Mathematics Ability

**Figure 2.**
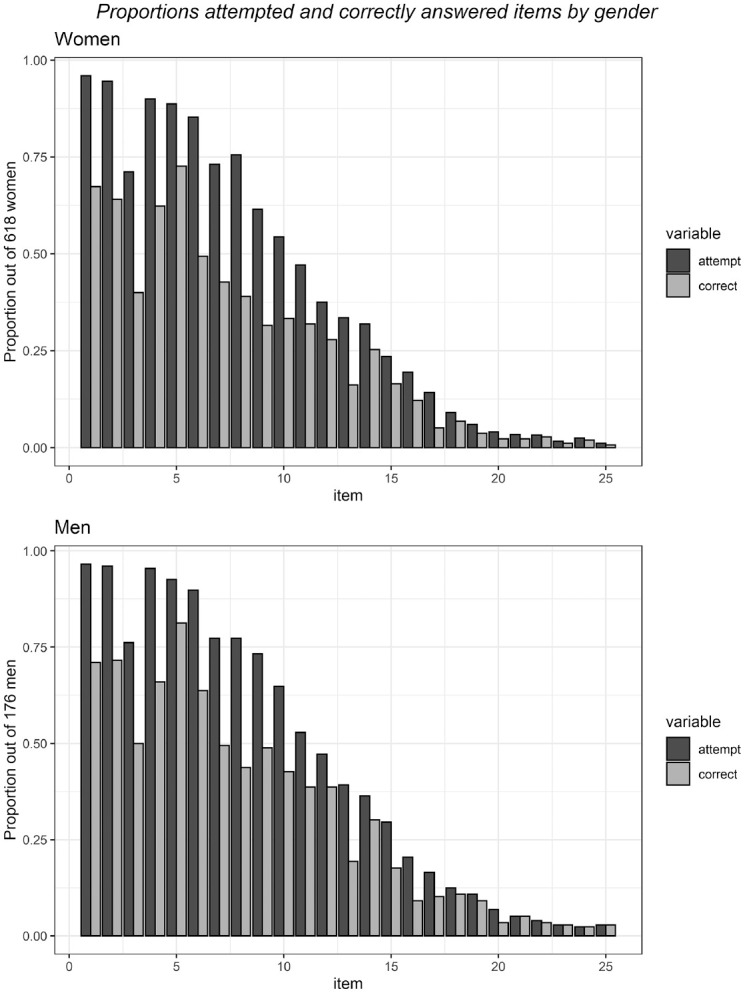
Proportions of the Number of Attempted Versus Correctly Answered Items by Female and Male Respondents.

**Figure 3.**
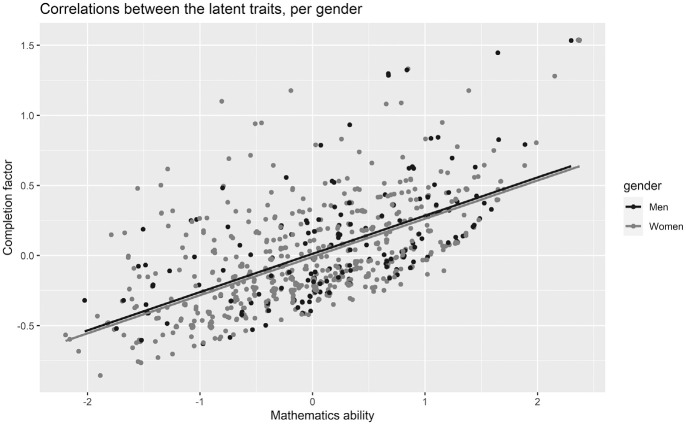
Correlations Between the Estimated Latent Trait Scores for Mathematics Ability and for the Completion Factor, per Gender.

In the first model, we found a positive correlation of *r* = .36 between the completion factor and mathematics ability (see Table 3 for variances and covariances), indicating that more able participants also dropped out later from the test. We illustrate this correlation by plotting the proportions of attempted and correctly answered items for both men and women (see Figure 2), where the items toward the end of the tests are answered correctly by few respondents (but note that these plots are based on the observed data, not the estimated latent traits). In Figure 3, we represent the correlations between the latent traits for both men and women. Item difficulty parameters ranged from −1.87 to 0.58 (*M* = −0.64), highlighting that the mathematics test was relatively easy for the university students.

**Table 3.** Estimated Variances and Covariances of the Latent Traits, for Models 1 to 3.

Data set	Parameter	Model 1	Model 2	Model 3
University	Variance of latent trait mathematics ability	$\sigma_{\theta_{1}}^{2}$ = 1.08	$\sigma_{\theta_{1}}^{2}$ = 1.06	$\sigma_{\theta_{1}}^{2}$ = 1.08
	Variance of latent trait completion factor	$\sigma_{\theta_{0}}^{2}$ = 0.47	$\sigma_{\theta_{0}}^{2}$ = 0.57	$\sigma_{\theta_{0}}^{2}$ = 0.49
	Covariance between latent traits	$\sigma_{\theta_{1},\theta_{0}}$ = 0.26	$\sigma_{\theta_{1},\theta_{0}}$ = 0.26	$\sigma_{\theta_{1},\theta_{0}}$ = 0.26

### Model 2: Gender Differences in Mathematics Ability and the Completion Factor

In Model 2, we estimated the potential gender gap on both latent traits. For both data sets, Model 2 did not converge with the preregistered settings, and we therefore increased the number of burn-in iterations to 100,000. Details on the convergence of the reported models can be found in Appendix A. On the sum scores, we found gender difference on both the total number of correctly answered (Cohen’s *d* = −0.33, 95% confidence interval [CI] [−0.50, −0.16]) and the number of attempted items (Cohen’s *d* = −0.22, 95% CI [−0.39, −0.06]). Women underperform compared with men on both sum scores, and on the total attempted scores we find a larger gender gap on the total correct scores.

**Figure 4.**
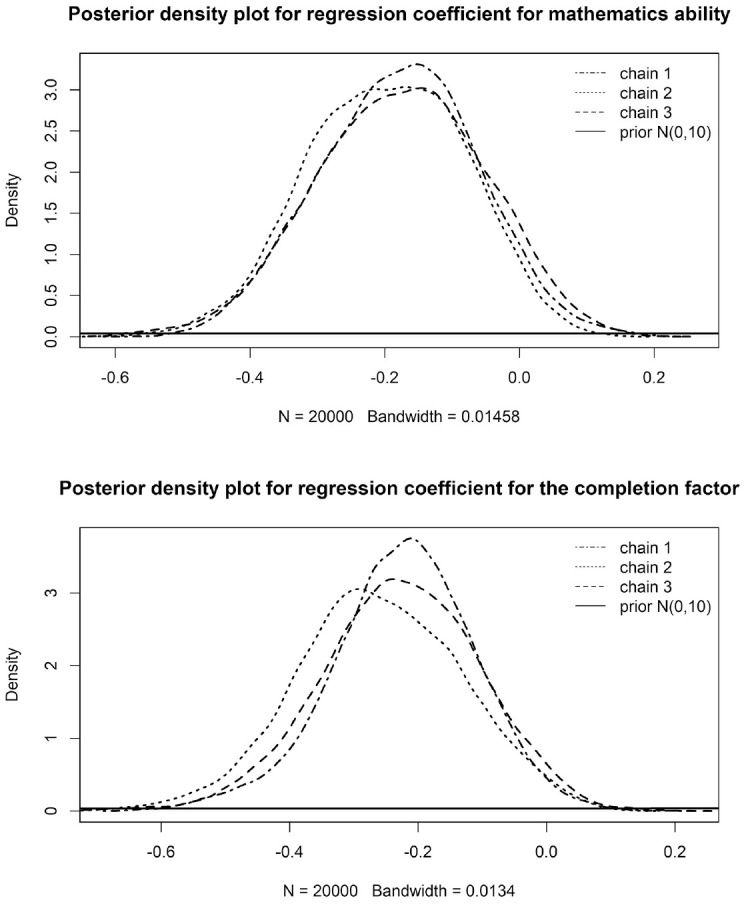
Posterior Density Plots for Both Regression Coefficients Beta. Beta1 Represents the Gender Gap on Mathematics Ability, and Beta2 the Gender Gap on the Completion Factor.

Again, we found a positive correlation between the completion factor and mathematics ability (*r* = .34), indicating that more able participants also dropped out later in the test. Item parameters changed slightly when including gender as a covariate, with mean difficulty being estimated slightly lower under this model compared with the estimates found under Model 1 (*M* = −0.78), and difficulty parameters ranging from −2.03 to 0.45. For mathematical ability, we did not find a gender gap, β_1_ = −0.18 (95% HPDI [−0.42, 0.04]), indicating that men and women did not differ in their scores on the latent trait mathematics ability. For the completion factor, however, we observed a gender gap favoring men, β_2_ = −0.24 (95% HPDI [−0.49, −0.004]), meaning that women attempted fewer items than their men. Posterior densities are displayed in Figure 4. Note that these effects are standardized and partly contradict what can be observed based on sum scores. Namely, here, the gender gap is larger on the completion factor than mathematics ability.

### Model 3: No Effect of Stereotype Threat on Mathematical Ability and the Completion Factor

In Model 3, we modeled the main effect of the stereotype threat condition on the two factors, and again found a positive correlation between completion and mathematics ability (*r* = .36, see Table 3). Difficulty parameters ranged from −1.89 to 0.57 (*M* = −0.64). Including the stereotype threat covariate did not change parameters substantially, compared with those found under Model 1. The effect of stereotype threat on both mathematics ability, γ_1_ = −0.02 (HPDI 95% [−0.21, 0.17]), and the completion factor, γ_2_ = −0.10 (HPDI 95% [−0.30, 0.09]), were close to zero.

### Additional Exploratory Analysis: Simple Effect of Stereotype Threat for Women

Because Model 4 which included an interaction between gender and stereotype threat failed to converge, we ran an (unregistered) exploratory model that included the data from women only and focused on comparing the stereotype threat conditions. Using the preregistered settings (see Appendix A), this additional model did converge and yielded results similar to those that also included the data for men (Model 3). For women only, we did not observe a stereotype threat effect on either one of the two latent traits, γ_1_ = −0.04 (HPDI 95% [−0.24, 0.15]) for mathematical ability and γ_2_ = −0.11 (HPDI 95% [−0.32, 0.11]) for the completion factor. The correlation remained positive, at *r* = .38.

## Discussion

Using data from a large registered replication study on stereotype threat (Stoevenbelt et al., in principle accepted), we investigated whether the observed gender gap on speeded mathematics tests can be (partly) explained by gender differences in test-taking strategies adapted by women and men under time limits. Furthermore, we assessed whether women in a stereotype threat condition are affected by a so-called double disadvantage with respect to their performance, due to potential additive effects of their gender identity and the stereotype threat manipulation. Concretely, using a Bayesian two-dimensional IRT model (Glas & Pimentel, 2008), we studied the relationship between the completion factor and mathematical ability, and any gender differences in both factors. Besides estimating the correlation between mathematical ability and the completion factor, we investigated whether there was a gender gap, an effect of experimental condition (stereotype threat), or an interaction between these two covariates, by including these covariates in subsequent analyses.

We found a small positive correlation (*r* = .36) between the two latent traits, indicating that more able test takers attempted more items. These results imply that missing data in stereotype threat research—and, by extension of argument, in other contexts perhaps as well—should not be ignored and should preferably be considered when analyzing data that were collected under time limits. If not accounted for, missing data can potentially lead to biased parameter estimates (Glas & Pimentel, 2008), such as underestimated item difficulties, and, in turn, could lead to unfairness in psychometric tests.

We did not find a simple effect of stereotype threat among women, indicating that neither latent factor was affected by the stereotype threat manipulation. Thus, we did not find any evidence for our hypothesis based on earlier work (Flore, 2018) that stereotype threat affects the test-taking strategies of threatened female test takers. In other words, we did not document any potential double disadvantage for women in the stereotype threat condition. We do note that the data collection of Stoevenbelt et al. (in principle accepted) was delayed due to COVID-19 and is still ongoing, and hence that results and conclusions with respect to stereotype threat effects may change once more data has been collected and analyzed. The current results align with additional recent findings in the literature on stereotype threat research showing that the stereotype threat effect is not as robust as thought before. As it stands, results based on replications and registered reports, with larger studies showing null effects, provide little to weak support for stereotype threat theory (Agnoli et al., 2021; Finnigan & Corker, 2016; Flore et al., 2018; Gibson et al., 2014; Moon & Roeder, 2014). Moreover, meta-analyses highlighted evidence for publication bias, with positive findings being more likely to end up in the more easily accessible research literature than negative findings (Flore & Wicherts, 2015; Shewach et al., 2019; Zigerell, 2017). Consequently, published articles that found (large) stereotype threat effects might paint a biased picture of the actual strength of the true effect, partly due to publication bias and selective reporting (Simmons et al., 2011; Wagenmakers et al., 2012; Wicherts et al., 2016). We encourage fellow researchers to conduct large preregistered studies to avoid such pitfalls. It may still be viable to apply our proposed models to large stereotype threat data sets that do demonstrate a stereotype threat effect. However, to our knowledge, such a large data set, preferably originating from a preregistered study, is currently not available, and, thus, we are unable to extend the current account with another data set.

We did find a gender gap on the completion factor estimates, but not on mathematical ability, although differences between factors in gender gaps were relatively small (*d* = −0.33 vs. *d* = −0.22). However, these results highlight that the women in our sample differ in test-taking strategies from their male peers, which might have important implications for measurement under time limits if the aim is to provide an estimate of mathematics ability unaffected by how many items are completed. Thus, concluding that women have a lower mathematics ability than men is not only a biased conclusion under time limits, but may also yield an unfair advantage for men when the test is used to select, for example, students to an academic program.

For instance, in tests that aim to measure mathematics ability, a time limit could potentially enlarge the gender gap because of completion differences (particularly when easier items are place at the end of the test) and hence time limits could even introduce violations of measurement invariance (when it is defined with respect to mathematics ability) and unfairness in testing. Besides its relation with stereotype threat effects, the model by Glas and Pimentel (2008) could be applied more widely to timed tests measuring mathematics and other cognitive abilities to assess the undesired differential impact across groups of time limits.

Our approach could provide a method to study gender differences on other speeded tasks, such as cognitive or intelligence tests, in order to differentiate between gender differences that are due to participants’ ability or due to the completion factor of the participants. We found evidence for the fact that missing data in experimental research cannot be ignored, because data missingness is related to the main construct of interest within stereotype threat data (Glas & Pimentel, 2008). The IRT approach we utilized could be applied to speeded tests in general. Specifically, we encourage researchers to consider the impact of their design choices before data collection, to avoid potential bias in their conclusions due to speeded tests.

### Limitations

Considering the width of the highest probability density interval and the difficulties we faced with model convergence, we highly encourage replicating this evidence in another stereotype threat data set that might become available in the future (including but not limited to the additional labs contributing to the RRR). We note that the samples used in this study were exclusively collected in Western Europe, thus resulting in Western, educated, industrialized, rich, and democratic (WEIRD) samples. The university data comprise undergraduate college students. Stereotype threat has been shown to affect both high school and college students (Doyle & Voyer, 2016; Flore & Wicherts, 2015; Nguyen et al., 2003). However, differences were shown across regions (Picho et al., 2013) and may affect results.

Furthermore, we used an experimental data set that has been collected under a low-stakes setting for participants, who might have been less motivated to put effort into their work. Low effort may impact the validity of participants’ test scores (Wise & DeMars, 2005). During data collection, we aimed to keep participants engaged with their tests, and they were not allowed to leave early or to work on other tasks during the experiment. However, this testing setting does not necessarily generalize to a high-stakes testing situation, such as the SAT, and our results may, therefore, also not generalize to speeded high-stakes tests.

In the models we applied here, we ignored skipped items (items that were not attempted by a participant, rather than not reached due to the time limit). Consequently, this approach does not fully reflect the entire missingness process at hand. In our approach, the fact that an item is missing is solely attributed to the existence of a strict time limit. However, properties of individual items (e.g., complexity) might explain (intermediate) missingness as well. Defining speed as the number of reached items foregoes the opportunity to, for example, include response times as an indicator of participants’ effort (e.g., DeMars & Wise, 2010) or item complexity. Including some measure of effort may be fruitful, as, for example, differential guessing behavior has been linked to gender differences in item skipping (Ben-Shakhar & Sinai, 1991), and differential item functioning (DIF; DeMars & Wise, 2010), even though results are mixed (Liu & Wilson, 2009). However, not accounting for differences in effort may lead to biased gender comparisons (Ben-Shakhar & Sinai, 1991; DeMars & Wise, 2010; Liu & Wilson, 2009). Unfortunately, because the included tests were administered on paper, the current data sets cannot be used to expand the proposed models with response time measures. Future researchers may wish to assess potential effects of participant effort, for example, by examining mean response times (see also Fox & Marianti, 2016).

Skipping behavior can also be included in item response models when response times are not available. Holman and Glas (2005) have proposed an item response model for skipped items, and Debeer et al. (2017) have suggested IRTree models that encompass both missingness patterns. However, because the two-dimensional IRT model by Glas and Pimentel (2008) already caused convergence issues in our data, we argue that it would not be reasonable to attempt fitting these models presently, given that they are even more complex.

We only focused on experimental psychological research. Within this context, we argue that if gender differences are found in an experiment, researchers should be cautious when attributing such differences to differences in the construct of interest, rather than to design choices, such as speededness, to avoid drawing faulty conclusions. We focus on stereotype threat research, but note that our argument can be extended to all operational tests and experimental settings wherein time limits are imposed.

Furthermore, we assumed linear relationships for stereotype threat throughout. We note that stereotype threat has originally been theorized to only affect high-achieving students (Spencer et al., 1999; Steele, 1997). However, a recent meta-analysis of 31 stereotype threat experiments showed no evidence for the effect of stereotype threat being differentiated according to prior academic achievement (e.g., as reflected in SAT scores; Stoevenbelt et al., 2022). However, there may be some scenarios where the linearity assumption may not hold. For example, participants with an efficient test-taking strategy might be more affected by stereotype threat effects than those without a good strategy, because the latter group does not have the skill set to tackle the items in the first place (regardless of the manipulation). For example, allowing for more time on the SAT only benefited the highly efficient students, but not those who already demonstrated inefficient problem-solving methods (Bridgeman, Trapani, & Curley, 2004), because more able respondents omit fewer items (Pohl et al., 2014). We also note that stereotype threat may influence women in the experimental condition without affecting mean effects. If the lower-end and the higher-end of the score distribution of participants are equally affected, the mean score of this experimental group will not change (see Flore, 2018, for a discussion of counterbalanced moderation of ability in stereotype threat research) for a discussion of counterbalanced moderation of ability in stereotype threat research). If this occurs, groups may appear not to differ on measures as Cohen’s *d*, even though they do differ in their score distributions (Voracek et al., 2013). Finally, we note that studying differences in subgroups, for example, differences between highly achieving and lower achieving respondents, may be interesting. However, we argue that the current model on a subset of the included data set may result in difficulties. Specifically, a direct selection on the basis of, for example, observed total mathematics scores would distort the factor structure and lead to lack of invariance with respect to the overall sample and other subgroups (Muthén, 1989).

Furthermore, we assumed that mathematics ability reflects a unidimensional construct. All items were obtained from the quantitative section of the GRE, which has been shown to reflect a quantitative reasoning factor (e.g., Stricker & Rock, 1985). One could test this assumption, for example, by fitting a fully explorative factor model. However, we argue that results of this analysis may be interpretable for the included data sets as the dimensionality of the tests could be affected by the imposed time limits. If the mathematics test is not unidimensional, it may be fruitful to extend IRT models that can incorporate subscales (e.g., De La Torre & Patz, 2005) to account for time limits.

Moreover, in light of sample size limitations, we did not specifically test for gender differences in the item parameters or DIF with respect to gender or stereotype threat condition (see also Flore, 2018; Wicherts et al., 2005). Future work, with again larger data sets, could shed more light on the potential effect of gender and/or stereotype threat on the measurement parameters.

Even with a simplified version of the model and despite the relatively large sample size, we had trouble to ensure that the models we suggested converged. They required many thousands of MCMC iterations before convergence was reached. Moreover, the proposed models did not converge on the high school data, even after 100,000 burn-in iterations. Convergence issues may result from low reliability and a high amount of noise in the data. For the university sample, especially for the models including gender as a covariate, we encountered convergence issues, potentially due to the samples’ skewed sex ratio (78% women). These issues could also stem from the comparatively large amount of missing data (58% in the university data set), or the relatively small correlation between the two latent traits. Larger numbers of missing data result in less data and less information for the model to work with.

### Concluding Remarks

In summary, we investigated here one of the dimensions that may enlarge gender differences in time test performance: Missing data caused by time limits and gender differences in completion rates. As a secondary goal, we investigated whether stereotype threat may affect the completion factor, rather than mathematics ability proper. In the current sample, we did not recover a stereotype threat effect on the completion factor; consequently, stereotype threat did not seem to affect how participants attempted items. We also did not find a stereotype threat effect on the mathematics factor, which is in line with the preregistered analyses of sum scores as reported in the RRR itself. We call for more large-scale, open, preregistered studies, such that researchers can adequately investigate whether stereotype threat exists on the level of items (see also Flore, 2018). Furthermore, mathematics ability was positively related to the completion factor, indicating that the missing data that originated from the time limits cannot be ignored in both the analysis of and the conclusions based on the data. Men and women did differ in their latent trait scores on the completion factor, but not on the mathematics ability trait. If these results generalize to other tests that use time limits, it is important to consider as an important factor in gender gaps as it relates to the validity of the measures and fairness in testing. If the completion factor is ignored and only ability is studied, this may lead to test unfairness, violations of measurement invariance, and invalid group comparisons, as well as potentially DIF (Dorans et al., 1988; Lu & Sireci, 2007). We urge researchers to take these effects of time limits into account when analyzing their data or, ideally, already during the design phase of their experiments.

## Appendix A

### Model Convergence

#### Model 1: No Covariates

The effective sample sizes for all parameters were above 594 for the university data, Monte Carlo standard errors (MCSEs) below 0.01 for all parameters, and $\hat{R}$ values that reflect chain convergence were between 1.00 and 1.01 for the university data.

#### Model 4: Full Models

All Monte Carlo standard errors were below 0.015. However, based on trace plots, effective sample sizes, and $\hat{R}$ values, we concluded that the model did not converge. With 100,000 burn-in iterations, effective sample sizes were only above 160. The effective sample sizes were under 400 for most parameters of interest, namely, the difficulty parameters of Items 1 to 13, the regression coefficients of the main effect of the gender covariate (betas 1 and 2), the variance of the latent trait representing the completion factor, and tau1, totaling 17 non-converged parameters. $\hat{R}$ values were above 1.05 for 21 parameters, including all parameters listed above, giving further evidence for non-convergence. In addition, we observed $\hat{R}$ values above 1.05 for the difficulty parameter of Item 14, the regression coefficient (delta) of the interaction for the latent trait representing the completion factor (delta2), and the regression coefficients of the main effect of stereotype threat (gamma’s 1 and 2). $\hat{R}$ values that were over 1.05 ranged from 1.051 (difficulty parameter Item 14) to 1.21 (tau1).

#### Model 2: Gender Covariate

All effective sample sizes were above 283, with the lowest effective sample size for the regression coefficient of gender on the completion latent trait (beta2). For two other parameters, effective sample sizes were below 400: for the regression coefficient on ability (384, beta1), and for tau1 (304). However, even though effective sample sizes were low, trace plots and other indices indicate that the model did converge. All MCSEs were below 0.01, and $\hat{R}$ values ranged between 1.00 and 1.03.

#### Model 3: Stereotype Threat Covariate

The effective sample sizes for all parameters were above 685 for the university data. Monte Carlo standard errors (MCSEs) were below 0.01 for all parameters the model fitted on the university data, and $\hat{R}$ values were between 1.00 and 1.02 for the university.

##### Exploratory Results: Simple Effect of Stereotype Threat for Women Only

The effective sample sizes for all parameters were above 630 for the university data. Monte Carlo standard errors (MCSEs) were below 0.01 for all parameters the model fitted on the university data, and $\hat{R}$ values were between 1.00 and 1.02 for the university.

## Appendix B

### Item Analysis of the High School Data

As mentioned, the proposed models did not converge based on the preregistered settings. We increased the number of burn-in iterations (up to 150,000 iterations) and implemented more informative priors by replacing all normal priors with standard normal distributions. However, despite these adaptations, Model 1 did not converge. As an exploratory analysis, we estimated a Bayesian Rasch model, using 3 chains and 1,000 iterations per phase and similar priors as for our confirmatory models (for code, see: https://osf.io/j8aqc). We found difficulty parameters ranging from −1.89 to 1.02 (*M* = −0.63) for the items of the high school mathematics test. In general, we found that students found the items relatively easy, with eight items showing proportions of correct answers >75%, and that test reliability was relatively low (α = .59). Therefore, the data may contain too little variance or too much noise to fit our proposed models.
